# Human NK Cell Diversity in Viral Infection: Ramifications of Ramification

**DOI:** 10.3389/fimmu.2016.00066

**Published:** 2016-03-03

**Authors:** Dara M. Strauss-Albee, Catherine A. Blish

**Affiliations:** ^1^Stanford Immunology, Stanford University School of Medicine, Stanford, CA, USA; ^2^Department of Medicine, Stanford University School of Medicine, Stanford, CA, USA

**Keywords:** natural killer cells, lymphocyte diversity, mass cytometry, viral susceptibility, single-cell technology

## Abstract

Natural killer (NK) cells are a unique lymphocyte lineage with remarkable agility in the rapid destruction of virus-infected cells. They are also the most poorly understood class of lymphocyte. A spectrum of activating and inhibitory receptors at the NK cell surface leads to an unusual and difficult-to-study mechanism of cellular recognition, as well as a very high capacity for diversity at the single-cell level. Here, we review the evidence for the role of NK cells in the earliest stage of human viral infection, and in its prevention. We argue that single-cell diversity is a logical evolutionary adaptation for their position in the immune response and contributes to their ability to kill virus-infected cells. Finally, we look to the future, where emerging single-cell technologies will enable a new generation of rigorous and clinically relevant studies on NK cells accounting for all of their unique and diverse characteristics.

Natural killer (NK) cells were discovered in 1975 (1, 2) on the basis of their ability to selectively lyse leukemic cell lines. Originally called “null cells” because they were believed not to express cell surface receptors, they were eventually recognized as a third lineage of lymphocyte distinct from T and B cells (3). Recently, the role of NK cells as the founding member of a growing group known as innate lymphoid cells has also been appreciated (4).

Natural killer cells are a unique lineage with remarkable agility. They rapidly detect and destroy virus-infected, malignant, and stressed cells (5, 6). They are also the most poorly understood class of lymphocyte, due in part to their unusual and difficult-to-study mechanism of cellular recognition. The NK cell surface contains a spectrum of activating and inhibitory receptors (7). It is the integration and balance of signals from these receptors that determine a cell’s activation status (8).

This array of receptors also generates the opportunity for vast diversity in the NK repertoire. Examination of this diversity has been limited: studying a large number of parameters on a single cell is technologically challenging. Yet, it may be a critical functional feature of the NK cell repertoire.

In many of the ways in which immune cells are conventionally categorized, NK cells are intermediates. They borrow, share, and combine functional features of other cell types to form their own recognition paradigm. This helps to explain their enigmatic nature and argues that they occupy a unique evolutionary niche. Here, we review this recognition paradigm, with a focus on NK responses to viruses, and argue that single-cell diversity enhances their ability to fulfill this “middleman” role. We also discuss current and future studies, where single-cell technology will allow a much more detailed and nuanced dissection of the roles and promise of NK cells in the antiviral response.

## NK Cells have Lifetimes of Intermediate Length

**Figure 1:**
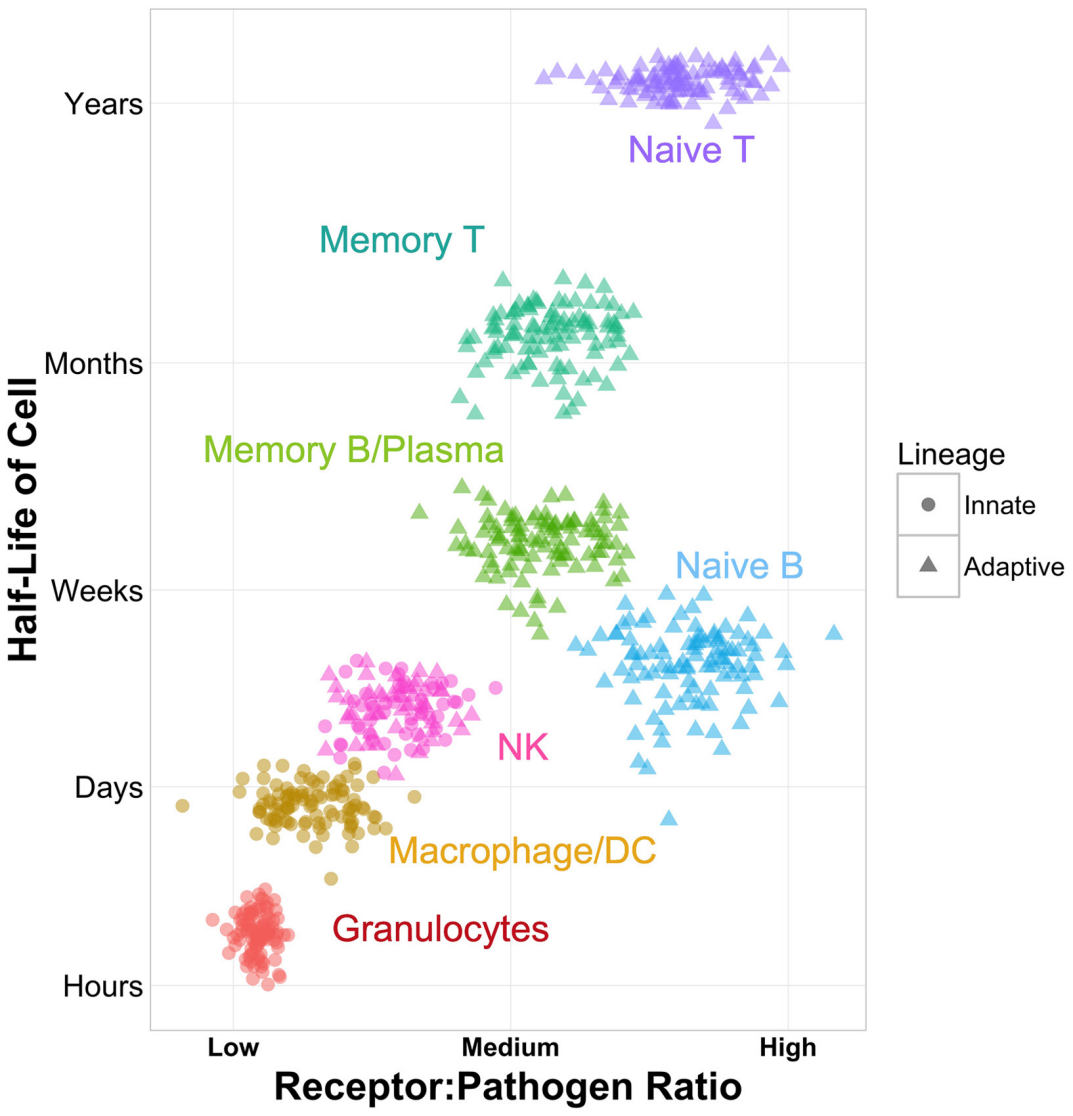
**Schematic of NK cells as immune intermediates in cell lineage, ratio of receptors to potential pathogens, and cellular half-life**. Half-life values are approximated based on published data (9–15).

In comparison to their classic innate and adaptive counterparts, NK cells take an intermediate position in estimates of cellular lifetime (Figure 1). In deuterium incorporation studies, human T cells have the longest estimated leukocyte half-lives, at 1–8 years for naive T cells and 1–12 months for memory T cells (9, 10). Estimated half-lives for CD27^−^ naive B cells, CD27^+^ memory B cells, and plasma cells are somewhat more limited, at 22, 11, and 40 days, respectively (11, 12). For NK cells, half-lives are shorter, estimated at 7 days (13). Yet, they still outlast their innate counterparts, with monocytes’ half-lives estimated at 71 h (14) and neutrophils’ reported between 5 and 90 h (15). It is important to note that only actively dividing cells incorporate deuterium, and thus these studies may not account for long-lived, non-dividing cells. They are also based entirely in peripheral blood; cell kinetics in tissues may follow completely different patterns. Yet, with these caveats in mind, these data suggest that NK cells fall midway on the spectrum of cellular lifetime. These estimates may also serve as a reasonable proxy for the amount of time they require to respond to viral infection.

## NK Cells are Intermediate in Their Mechanism of Recognition

Cells of the innate system typically recognize non-self entities through specific receptor–ligand interactions. The toll-like receptor (TLR) system and other pattern recognition receptors, including RIG-I-like receptors, NOD-like receptors, and C-type lectin receptors, are designed to recognize specific features of pathogens or other danger signals (16, 17). Recognition is fast and digital, having only on/off states. It triggers phagocytosis, the release of inflammatory cytokines, and regulation of downstream adaptive responses. With 10 TLRs identified in humans to date and other pattern recognition receptor classes in the same order of magnitude or fewer, the innate paradigm is characterized by a low ratio of receptors per potential pathogen (Figure 1).

While the innate system has evolved to recognize broad classes of pathogens through a limited set of receptors, the adaptive system takes the converse approach of employing a broad set of receptors that each recognize a very limited set of pathogens. In T and B cells, V(D)J recombination forms specialized, antigen-specific receptors. When triggered in combination with costimulation, these receptors initiate proliferation, cytotoxicity, production of antibodies, and release of cytokines. The total potential diversity of the human T cell receptor (TCR) is estimated at 10^15^–10^20^ sequences (18, 19). The adaptive system is therefore characterized by a high ratio of receptors to potential pathogens (Figure 1).

Natural killer cells take cues from both extremes. They express a spectrum of receptors, which can be either activating or inhibitory, through which they integrate signals to determine their activation status. These include killer immunoglobulin-like receptors (KIR), C-type lectin-like receptors, signaling lymphocyte activation molecule (SLAM) family receptors, and natural cytotoxicity receptors, among others (20). Unlike other cell types, NK activation is analog: increased activating or decreased inhibitory signals can tip the balance toward activation, but typically no specific receptor–ligand interaction is required. In fact, CD16 is the only receptor that has been shown to be capable of activating NK cells independently (21). In addition, NK cells are the only lymphocytes that can be activated purely through soluble signals. Proliferation, cytotoxicity, and a migratory phenotype can be induced by cytokines alone without specific antigen signal or presentation (22, 23).

Thus, the lifespan of NK cells, their mechanism of recognition, and in addition, recent evidence for memory in NK cell responses (24–26) suggest that they play a truly intermediate role between innate and adaptive responses (Figure 1).

## Single-Cell Diversity: A Logical Adaptation for Middlemen

The diversity in a T cell or B cell repertoire is captured in a single receptor. NK cells, by contrast, are diverse at a single-cell level. Each cell expresses a combination of receptors drawn from a vast potential pool. Our early estimates, based on 28 markers, place the total number of combinations in human peripheral blood on the order of 10^4^ (27). With deeper sampling, measurement in a greater diversity of tissues, and improvements in technology allowing increased dimensionality, this estimate will certainly increase. A modest increase to measuring 40 NK cell receptors on a single cell would yield 10^12^ theoretical combinations. In comparison, T cells have been estimated to have 2.5 × 10^7^ unique TCR sequences in humans (28). As mentioned above, their total potential diversity is estimated at 10^15^–10^20^ sequences (18, 19).

Thus, both theoretically and empirically, NK cells are highly diverse, but several orders of magnitude less so than T cells. Yet, T cell (and B cell) diversity is rigid, modifiable primarily by population-level expansion and contraction. The antigen-specific TCR or BCR, while optimizable via somatic hypermutation or affinity maturation, is pre-formed following exit from the bone marrow or thymus. For adaptive cells tasked with developing a slow but reliable memory response, it is a logical tradeoff to expend energy on selecting a vast but inflexible repertoire.

Natural killer diversity compensates for its lack of breadth with its flexibility, a logical evolutionary adaptation for cells that must act quickly. NK diversity can be modulated with vastly more agility and at many different levels. NK cells can up- or downregulate receptors to adjust repertoire diversity at the timescale of cellular processes rather than cellular division (29), one cell at a time rather than by population. Many NK cell receptors, especially the KIRs, are defined by vast population-level genetic diversity (30). This can be compounded at multiple levels: selective transcription and translation, post-translational modifications, protein trafficking, cytokine signaling, cell–cell interactions, and epigenetic modifications (31–33). NK cell diversity is thus much more responsive to both genetic and environmental determinants. This responsiveness may prove to be a critical feature for maintaining flexibility in the earliest lymphocyte response.

## NK Cells in Viral Susceptibility

Because of their uniquely centered position in the immune system, their associations with improved control of viral infections (34), and recent evidence of their potential for a memory response, much interest has been focused on whether NK cells can be harnessed as part of a strategy for the initial prevention of viral infection (Table 1). Prevention is an especially significant goal for chronic incurable diseases, such as HIV. Mechanisms by which NK cells could successfully prevent viral infections include localization of NK cells to barriers of viral entry, efficient cytotoxicity, swift production of IFN-γ for recruitment and activation of downstream adaptive responses, and production of β-chemokines that can block viral entry (35).

**Table 1:** **Evidence for the role of NK cells in the prevention of viral acquisition**.

Virus	Study population	Major finding	Total sample size	Reference
HSV-1	HSV-1-infected adults	The presence of KIR2DL2 or KIR2DS2 by PCR was associated with progression to symptomatic, as compared to asymptomatic, HSV-1 infection	131	(36)
HCV	HCV patients and healthy adult controls	RNA^+^ HCV patients had increased presence of KIR2DL2 or KIR2DS2 by PCR compared with self-limited RNA^−^ HCV patients	596	(37)
	Acutely infected HCV patients and healthy adults	NK cells from acutely infected HCV patients produced more IFN-γ and degranulated more than NK cells from healthy controls	39, 44	(38, 39)
	People who inject drugs with or without seroconversion or spontaneous clearance	Relative to chronically infected individuals, homozygosity for KIR2DL3 and its ligand HLA-C1 was more frequent in exposed seronegative individuals or those who spontaneously cleared the virus	1037, 305	(40, 41)
	Acute and chronic HCV patients, patients who naturally resolved infection, and healthy controls	During acute infection, fewer NKp30^+^, NKp46^+^, CD161^+^, and NKG2D^+^ NK cells were present in individuals who subsequently cleared than those who became chronically infected	57	(42)
	People who inject drugs with or without seroconversion, healthy adults	Higher anti-K562 cytotoxicity and higher NKp30 expression detected in exposed uninfected individuals	33	(43)
	People who inject drugs with or without seroconversion, healthy adults	Presence of KIR2DL3^+^NKG2A^−^ NK cells was associated with protection from productive HCV infection	114	(44)
Chikungunya	Chikungunya patients, healthy controls	NK cells from acutely infected chikungunya patients become activated and expand early in infection	55, 143	(45, 46)
EBV	EBV-college students	Increased CD56^dim^NKG2A^+^CD57^+^NK cells detected in peripheral blood during acute infectious mononucleosis	18	(47)
CMV	Solid organ transplant recipients, bone marrow transplant recipients	CD57^+^NKG2C^hi^ NK cells preferentially respond during CMV reactivation	140, 65	(48, 49)
HIV	South African women: cases acquired HIV, matched controls did not	Decreased pre-infection IFN-γ responses to autologous infected CD4^+^ T cells were associated with increased acquisition risk	60	(50)
	South African women: cases acquired HIV, matched controls did not	Increased pre-infection NK activation (higher HLA-DR and lower CD38) was associated with increased acquisition risk	81	(51)
	Exposed uninfected intravascular drug users, seroconverters before or after seroconversion, unexposed controls	NK cells from exposed uninfected intravascular drug users showed greater lytic activity and produced more cytokines in response to cell lines than unexposed controls or seroconverters before or after seroconversion	75	(52)
	Kenyan women: cases acquired HIV, matched controls did not	Increased pre-infection NK diversity correlated with increased acquisition risk	36	(53)

Directly studying the immune correlates of human viral susceptibility is challenging. It requires either tracking uninfected people over long periods of time, a difficult logistical hurdle, or infecting people in a controlled environment, an ethical quandary. Several approaches have therefore been used to provide indirect evidence for the importance of NK cells in human viral susceptibility.

First, much has been learned from studies of immunodeficient subjects with deficiencies in NK cell frequency or function. In addition to several mutations known to exert their effects on NK cells in relative isolation, at least 46 primary immunodeficiencies are also associated with NK cell defects. These subjects’ overarching and unifying feature is the unusual susceptibility to herpesviruses (54), especially HSV-1, EBV, VSV, and HPV. These findings suggest that NK cells play a non-redundant role in preventing the establishment of these infections.

Second, many studies identify NK-relevant genetic correlates associated with disease-afflicted individuals versus healthy controls. These retrospective studies are not direct measures of acquisition probability, but do provide evidence of possible associations. The presence of KIR2DL2 and KIR2DS2 has been associated with progression to infection in both HSV-1 (36) and HCV (37).

A third approach has been to examine the characteristics and strength of NK responses during acute infection and sometimes draw correlations with disease outcome. In these studies, blood is typically drawn at the time of clinical presentation. This approach provides an imprecise approximation of the earliest events in infection but has the distinct advantage of allowing functional immunological studies. These studies have identified several early-stage markers of NK cell activation in viral infection. In HCV, KIR2DL3 in combination with HLA-C1 at both the genetic and cellular level has emerged as an important correlate of protection from infection (40, 41, 44). In addition, NKG2C^+^ NK cells have been shown to specifically respond to CMV reactivation in transplant recipients (48, 49).

A fourth type of study measures the immune activity of individuals who remain seronegative despite behavior that grants high probability of viral exposure, providing clues as to what is assumed to be an effective protective immune response. The primary issue with these studies is that this form of resistance is not fully understood, and the highly exposed immune state of these individuals (typically sex workers or people who inject drugs) may not be representative of other routes of acquisition.

Finally, a handful of studies, so far only in the HIV field, have enrolled and tracked large cohorts at risk of infection, banking blood samples before and after detection of infection, and performed functional studies on pre-infection samples. While impressive in scope, their conclusions have unfortunately not been entirely clear. While the ability of NK cells to secrete IFN-γ in response to HIV-infected cells was associated with decreased risk of infection (50), generalized NK cell activation (as measured primarily by expression of CD38 and/or HLA-DR) was associated with increased risk of HIV acquisition (51). Thus, depending on its nature, NK cell activation has been both positively and negatively associated with increased risk of HIV acquisition.

Our recent study took a different approach. In addition to measuring activation status and individual markers, we calculated a diversity score for the NK cell repertoire of each donor before the onset of HIV infection. We showed that no single marker was predictive of HIV acquisition risk, but that higher NK cell diversity was associated with increased risk of HIV acquisition in a cohort of Kenyan women (53). This raises the intriguing possibility that NK repertoire diversification is detrimental to the NK cell response. As it represents an apparent state of ramification and inflexibility, high NK diversity may signal a higher risk of exposure and/or less resistant state of immunity.

All of these studies involve clinical cohorts that are limited in size. The brute-force approach of obtaining additional validation cohorts will be a necessary step in bolstering these results. However, targeted single-cell approaches that incorporate leading edge technologies and account for the increasingly appreciated diversity of the NK repertoire are in rapid development. These approaches hold great promise in bringing a new level of understanding to the role of NK cells in viral susceptibility.

## New NK Cell-Focused Approaches in Single-Cell Technology

Innovative microchip-based approaches have begun to uncover broad diversity in single-cell function. In a single-cell tracking system, the distribution of average kill times on a per-NK-cell basis was skewed, with a few “serial killers” performing the majority of the killing (55, 56). Furthermore, increased cytotoxicity has been shown to result from simultaneous interaction of NK cells with multiple targets (57) as well as IL-2 activation (23). NKG2A^+^ NK cells have also been shown to be superior to NKG2A^−^ NK cells in terms of dynamic migration, conjugation, spreading, and killing. “Serial killers” were also more common in this population (58).

Natural killer cell-focused mass cytometry, which has been pioneered by our group and others (27, 53, 59, 60), also holds great promise in further defining the role of NK diversity in viral acquisition at a single-cell level. This technology will be especially powerful when used in conjunction with humanized mice (61), presenting the next frontier in detailed tracking of all stages of the human NK cell response to viral infection.

Furthermore, single-cell dissection of functional diversity, especially in the context of a viral response, will greatly improve understanding of the processes by which NK cells may contribute to the prevention of infection. The generation of more single-cell data will also help to alleviate the statistical issues associated with small sample sizes in clinical studies.

Many questions remain about the functional consequences of NK cell diversity. Does a differentiated NK cell respond better and faster when re-encountering its initial stimulus? What is the spectrum of antigens that can drive NK cell diversification? Does a diverse NK cell repertoire correspond to a less diverse T and B cell repertoire? In addition, most studies have focused only on peripheral blood, but tissue-specific functions of NK cells are increasingly being appreciated (62).

Together, single-cell studies offer great promise in defining the scope of the human NK repertoire and its significance in the context of viral infection. This understanding will be essential in order to optimally harness the NK cells in the next generation of NK cell-based vaccines and therapeutics.

## Author Contributions

CB and DSA wrote the manuscript, and approved it for publication.

## Conflict of Interest Statement

The authors declare that the research was conducted in the absence of any commercial or financial relationships that could be construed as a potential conflict of interest.
